# Gender, smoking and tobacco reduction and cessation: a scoping review

**DOI:** 10.1186/s12939-014-0114-2

**Published:** 2014-12-12

**Authors:** Joan L Bottorff, Rebecca Haines-Saah, Mary T Kelly, John L Oliffe, Iris Torchalla, Nancy Poole, Lorraine Greaves, Carole A Robinson, Mary HH Ensom, Chizimuzo TC Okoli, J Craig Phillips

**Affiliations:** Institute for Healthy Living and Chronic Disease Prevention, University of British Columbia, 3333 University Way, Kelowna, BC V1V 2V7 Canada; Faculty of Health Sciences, Australian Catholic University, Melbourne, Australia; School of Nursing, University of British Columbia, 302-6190 Agronomy Road, Vancouver, BC V6T 1Z3 Canada; Centre for Health Evaluation and Outcome Sciences, Room 620, 1081 Burrard Street, Vancouver, BC V6Z 1Y6 Canada; British Columbia Centre of Excellence for Women’s Health, E311 – 4500 Oak Street, Box 48, Vancouver, BC V6H 3N1 Canada; School of Nursing, University of British Columbia, 3333 University Way, Kelowna, BC V1V 1V7 Canada; Faculty of Pharmaceutical Sciences, University of British Columbia, 2405 Wesbrook Mall, Vancouver, BC V6T 1Z3 Canada; College of Nursing, 315 College of Nursing Building, University of Kentucky, Lexington, KY 40536-0232 USA; RGN 3249A, School of Nursing, University of Ottawa, Guidon Hall, 451 Smyth Road, Ottawa, ON K1H 8M5 Canada

**Keywords:** Tobacco, Smoking cessation, Gender, Gender relations, Gender analysis, Scoping review

## Abstract

Considerations of how gender-related factors influence smoking first appeared over 20 years ago in the work of critical and feminist scholars. This scholarship highlighted the need to consider the social and cultural context of women’s tobacco use and the relationships between smoking and gender inequity. Parallel research on men’s smoking and masculinities has only recently emerged with some attention being given to gender influences on men’s tobacco use. Since that time, a multidisciplinary literature addressing women *and* men’s tobacco use has spanned the social, psychological and medical sciences. To incorporate these gender-related factors into tobacco reduction and cessation interventions, our research team identified the need to clarify the current theoretical and methodological interpretations of gender within the context of tobacco research. To address this need a scoping review of the published literature was conducted focussing on tobacco reduction and cessation from the perspective of three aspects of gender: gender roles, gender identities, and gender relations. Findings of the review indicate that there is a need for greater clarity on how researchers define and conceptualize gender and its significance for tobacco control. Patterns and anomalies in the literature are described to guide the future development of interventions that are gender-sensitive and gender-specific. Three principles for including gender-related factors in tobacco reduction and cessation interventions were identified: a) the need to build upon solid conceptualizations of gender, b) the importance of including components that comprehensively address gender-related influences, and c) the importance of promoting gender equity and healthy gender norms, roles and relations.

## Introduction

Tobacco reduction and cessation (TRC) are among the most important health promoting changes that men and women who smoke can undertake to reduce their risk of lung, breast, and cervical cancers and cardiovascular disease [1]. Smoking cessation is also an important mechanism to protect the health of others who might be exposed to second- and third-hand smoke and reduce the likelihood of influencing children, partners and peers to smoke [2] Although general smoking prevalence in Canada, the USA and many other Western countries decreased steadily from over 60% in 1965 to 16.7% in 2010 [3,4], declines have stalled and tobacco use remains high among many subgroups, in particular gendered groups who may be socially and/or economically disadvantaged. For example, low-income mothers [5], men who work in construction, trades or “blue collar” occupations [6], and men and women who identify as lesbian, gay, bisexual, and transgender (LGBT) [7] have smoking rates that far exceed the overall population prevalence. For those wanting to quit, long-term abstinence rates following smoking interventions that combine counseling with medication decrease to 20-35% after 12 months [8,9], pointing to the need to develop and modify TRC programs.

While a multidisciplinary body of research addressing gender-related influences has slowly emerged, the vast majority of that work is descriptive. Among other foci, there remains the need to transition gender-focused research toward evaluated TRC interventions targeted to high-risk subgroups [10]. Given this, it is critical to clarify the theoretical and methodological interpretations of gender in the context of tobacco research. In this article, we review the evidence regarding gender-related influences on tobacco smoking and cessation, and propose some ways forward by which gender may be interpreted and better incorporated into TRC interventions.

### Current approaches in tobacco interventions

In the medical community, tobacco dependence is typically viewed as a “chronic disease” [11,12]; and classified as a mental illness, tobacco use disorder, in the 5^th^ edition of the Diagnostic and Statistical Manual of Mental Disorders (DSM-5) [13]. By compartmentalizing smoking as a physical dependence and addiction, this biomedical definition neglects the social contexts that promote and prohibit smoking [14,15]. While biomedical approaches to tobacco use comprise part of the picture, they fail to integrate findings from the social sciences addressing gender-related influences on smoking as linked to both women’s and men’s social roles, identities, and relationships, and the influence of gender as a determinant of health and source of health inequity.

The approach to TRC interventions in the past two decades has often promoted the use of treatment matching strategies, whereby subgroups of people who smoke receive different treatments depending on individual vulnerabilities to treatment failure or relapse, and adapted to individual needs [16]. Treatment matching strategies have three major foci, often used in combination: 1) stepped-care programs where all people who smoke receive initial low-intensity interventions, and those who do not succeed are stepped-up to more intensive interventions until they succeed [17]; 2) stage-matched programs where intervention components are individualized to match the smoker’s current stage of change [18]; and 3) tailored interventions which are modified with existing standard treatments designed to address the needs of certain at-risk smoker subgroups (e.g., people that smoke with medical or psychiatric diagnoses such as cardiovascular diseases, diabetes, depression, or schizophrenia) [19]. Further, in the 53 Cochrane reviews of interventions to help people quit tobacco [20] “specific groups of smokers” were also identified by diagnoses, such as schizophrenia, depression or other substance use, and gender-related factors were not disaggregated or explicitly considered in interventions.

### Conceptualizing gender for TRC

Gender is defined as a multidimensional, social construct that refers to the processes by which we enact our belonging to various categories of being a woman, man, or transgendered person. The concept of gender is culturally and socially specific and changes over time. In this scoping review, we explore gender from a social constructionist perspective [21,22] which includes: socially prescribed roles and responsibilities (the gendering of social norms that influence who smokes and how, where, and when); aspects of individual identity and alignment with femininity or masculinity (smoking as “feminine rebellion” or “masculine cool”); gender relations (the tobacco-related micro-interactions among and between women and men that contribute to the construction, maintenance or contesting of gender); and institutional gender (the ways in which social organizations such as the tobacco industry or governments construct gender) [23]. In addition to expanding the concept of gender, awareness of the diversity and plurality of gender is also significant for tobacco research and interventions because subgroups of individuals, such as LGBT communities, can show a high prevalence of tobacco use [10].^a^

The tobacco industry has systematically and consistently marketed cigarettes from a gendered perspective, (representing women’s smoking as a means to enhanced femininity, heterosexual appeal, or rebellion [24] and men’s smoking as a means to masculine strength, manliness and freedom [25]), continuing to refine ads for target gender groups [26]. In comparison to the tobacco industry’s long-time exploitation of femininity and masculinity to sell cigarettes, the consideration of gender-related influences on TRC is a relatively recent development in tobacco control and health behavior research [27,28].

National guidelines for treating tobacco dependence [11,12] have not attended to the multifaceted elements of gender. In fact, it has been argued that clinical frameworks and guidelines for treating tobacco dependence [11,12] are somewhat gender neutral and/or gender blind despite burgeoning evidence that gender (as it intersects with culture, class and age) influences tobacco use [29,30]. For instance, women have been labeled as a “special population” in treatment guidelines and presumed to benefit from the same interventions as men. Likewise, despite men’s long historical connection to tobacco use and dependence, men receive no specific mention or discussion in treatment guidelines. Tobacco dependence treatment guidelines appear to reflect the notion of gender as, primarily, a medical and biological construct affecting women’s health; as a result, interventions for women are considered gender-specific within the context of enhancing interventions with components that address sex/biology and reproductive health (i.e., pregnancy, fertility, osteoporosis, hormones and other medical concerns).

A systematic review identified 39 intervention studies developed specifically for women [31]; however, among these interventions, the concept of gender was often limited to women’s assignment to subgroups based on a single characteristic (e.g., abnormal pap smears, the menstrual cycle, depression, or sedentary lifestyle). An understanding or conceptualization of gender as a complex set of influences connected to social power relations and/or social constructs of femininity was absent. Another systematic review identified 11 intervention studies targeting men [32]; however, among these interventions only two included treatment components tailored specifically for men (expectant fathers and gay men) [33,34]. The other interventions delivered standard (non-gendered) TRC treatments in *settings* providing health and social services specifically to men.

The purpose of this scoping review is to take stock of developments in the consideration of gender-related influences in smoking and TRC. Our objectives were to: a) examine how gender-related influences have been taken up in the wide-ranging literature on women and men’s smoking and tobacco reduction and cessation, 2) describe current knowledge related to gender-related influences on smoking and the associated implications for TRC interventions, and 3) propose principles to guide the development of gender-sensitive and gender-specific TRC interventions.^b^

## Methods

We conducted a scoping study [35,36] of the published literature to capture the relevant articles and books addressing smoking and TRC from the perspective of three aspects of gender: gender roles, gender identities, and/or gender relations. Scoping reviews allow researchers to map a specific field of research to determine its breadth and depth, summarize an area of research, identify gaps, and analytically assess the state of the literature. Unlike systematic reviews, scoping reviews do not assess the quality of the individual studies [35]. Adhering to the Arksey and O’Malley methodological framework for scoping reviews refined by Levac, Colquhoun, & O’Brien [35], we employed an iterative approach for determining study inclusion and data extraction; thematic qualitative analyses of the results; implications for policy; and the utilization of a consultative process to obtain additional insights [35]. Inclusion criteria were: items published in English after 1990 that addressed tobacco use or reduction and cessation within the context of gender roles, gender identity, or gender relations. We excluded items that conflated gender with sex (e.g., gender differences with sex differences); confined the concept of gender to biological categories of females and males; or focused on tobacco use prevalence, epidemiology; or demographic characteristics of tobacco users and their smoking patterns without consideration of gender-related influences.

In addition, we developed an original four step strategy to identify the relevant literature (Table 1). First we performed electronic searches in the databases CINAHL, PsychInfo, PubMed Sociological Abstracts, and EMBASE, for all citations occurring since 1990 using the search terms: smoking cessation, cigarette smoking, gender relations, gender identity, gender roles, masculinity, and femininity. After excluding citations that did not meet the inclusion criteria, we identified 13 articles that investigated smoking from at least one of the three aforementioned aspects of gender. Second, we manually “hand searched” the reference lists of these 13 articles (i.e., ancestry searching or searching backwards), and this yielded 31 relevant published articles. Third, we carried out a descendancy search in Google Scholar (i.e., searching forwards) to identify more recently published articles that may have cited any of the 13 retrieved articles from the aforementioned step one search strategy. This strategy produced an additional 13 articles for inclusion. Lastly, the authors and tobacco experts from our larger research team were polled for possible additions to the list of 57 [37] articles generated by Steps 1 to 3. This generated 23 new references including books, in press articles and peer-reviewed journal articles addressing tobacco and gender that we “hand-picked.” In total, 80 articles met our scoping review criteria.^c^

**Table 1:** **Scoping review process**

**Step**	**Resulting references**	**References identified alphabetically by author(s)**
1. Database Keyword Search	13	Bottorff et al. 2006a, 2006b,2009, 2012a; Gage, Everett, & Bullock, 2007; Gilbert 2007; Greaves et al. 2010; Haines et al. 2010a; Johnson et al. 2009; Oliffe et al. 2010; Roberts 2006; Tan 2011; Tinkler 2003
2. Ancestry Search	31	Amos & Haglund 2000; Anderson, Glantz, & Ling 2005; Barraclough 1999; Bottorff et al. 2005a, 2005b; Bottorff et al. 2010a; Cortese & Ling 2011; Cullen 2010; Dutta & Boyd 2007; Greaves, Kalaw & Bottorff 2007; Greaves & Hemsing 2009a; Haines, Poland & Johnson 2009; Kohrman, 2007; Macdonald & Wright 2002; Michel & Amos 1997; Morrow et al. 2002; Morrow & Barraclough 2003a, 2003b, 2010; Nawi, Weinehall & Öhman, 2007; Nichter et al. 2006, 2009; Oliffe et al. 2008; Oliffe, Bottorff & Sarbit 2012a; Pachankis, Westmass & Dougherty 2011; Rugkasa et al. 2003; Tinkler 2001a, 2001b; Toll & Ling 2005; Wearing & Wearing 2000;Westmass, Wild & Ferrence 2002
3. Descendancy Search	13	Alexander et al. 2010; Bottorff et al. 2010b, 2010c; Burgess, Fu & Van Ryn, 2009; Cook 2008; Gage, Everett & Bullock, 2011; Greaves & Jategaonkar 2006; Greaves & Hemsing 2009b; Haines et al. 2010b; Hemsing et al. 2012; Jackson & Tinkler, 2007; Robinson et al. 2010; White, Oliffe & Bottorff 2012a
4. Manual Inclusion	23	Amos et al. 2012; Bottorff et al. 2012b; Cook 2012; Ernster et al. 2000; Graham 1994; Greaves 1996; Greaves & Tungohan 2007; Haines-Saah 2011; Hunt, Hannah & West 2004; Mao, Bristow & Robinson 2012; Mao 2013; Nichter et al. 2010; Oaks 2001; Okoli et al. 2011; Oliffe, Bottorff & Sarbit 2012b; Rudy 2005; Schmitz 2000; Tinkler 2006; Wakefiled et al. 1998; White, Oliffe & Bottorff 2012b, 2012c, 2013a, 2013b
**Total:**	**80**	

Once the final list of studies was complete, we undertook a process of extraction, analysis and categorization for each of these items. We developed a data extraction key and a standardized table in order to systematically assess how each journal or article defined and theorized gender. Extraction sub-headings included entries describing the references’ focus, the methods and measures employed (e.g., qualitative, historical analysis, etc.), the language and terms used to describe gender (i.e., women, men, feminism, masculinity, etc.), and also noted whether or not there was a consideration of sex-related factors. We also used this table to determine whether the focus on gender in the text was implicit or assumed, or made explicit with clear and identifiable definitions and/or theories of gender used within the text. We then grouped each of the 80 articles into broad categories, based on the topics or themes we observed across this set of articles: gender relations and tobacco (n = 24); women’s smoking and gender identities, roles and norms (n = 25); men’s smoking and gender identities, roles and norms (n = 21); and gender issues in tobacco and policy (n = 10).

Members of the multidisciplinary research team participated in analyzing the articles, extracting and charting the data, and assigning each item to a category. After the completion of data extraction, two of the authors (MK, RH-S) undertook the analysis of items by assigning each article to one of four thematic categories. Below we present a critical analysis and qualitative synthesis of gender issues and tobacco as seen within each of these four categories.

### Results

The majority of articles in our review were from the domains of nursing science and social science research on smoking and health. As a set, the 80 articles were diverse and far-reaching in terms of their foci, methods, and theoretical orientations. The majority of the items reported on the results of empirical studies (n = 59), employed qualitative methods (n = 53) and primarily used one-to-one interviews and focus groups with people who smoke or who were engaging with cessation. Six articles reported findings from quantitative (n = 4) or mixed method (n = 2) studies, using survey instruments or psychological measures to assess the relationship between gender and smoking at an individual level. Eleven articles comprised literature reviews of varying scope, and two articles were based on the development of father-specific interventions [38,39]. There were 8 items, books or book chapters, which looked broadly at women’s and men’s smoking, and gender identities in the social and cultural history of tobacco use.

For each of these thematic categories, we describe the general features of the assigned articles, how they define and address gender issues, their theoretical approach to gender, and their potential implications for gender-sensitive and gender-specific TRC and interventions.

### Women’s smoking and gender identities, roles and norms (n = 25)

#### Description

There were 25 items addressing women’s smoking and feminine identities, roles or norms that met the criteria for inclusion in our review. These articles were published between 1994 and 2012.

In this category, 11 of the articles and books focused retrospectively on gender norms and the social and cultural history of women and smoking in the context of 20^th^ century Canada, the United States and the United Kingdom [40-50]. Two groups of scholars looked specifically at how the tobacco industry has used gendered imagery to market cigarettes to women in the late 20^th^ century [24,51]. These works highlighted the relationship between women’s smoking and the social context of gender inequality and changing gender roles. Nine empirical articles addressed gendered social norms and women’s smoking vis-à-vis a more contemporary context [52], with most centred on the gendered experiences of adolescent and young adult women who smoke [53-57], and considerations of how gender shapes young women’s responses to cessation and prevention campaigns [57-59].

There were two items that adopted a critical stance towards the gendered politics of smoking during pregnancy and by women who are mothers. Oaks’ [44] book interrogated public health’s use of “fetus-centric” rhetoric and approaches to smoking cessation that neglect the health of women beyond gestation and their roles as mothers. Likewise, Graham’s monograph established a more complex relationship between gender and tobacco use, illustrating how smoking by low-income, lone-parent women is a function of their living in poverty and experiencing social marginalization [60].

Almost all of the retrieved items employed historical (archival research, industry document analysis) or qualitative methods (interviews and focus groups) to address women’s smoking. A handful of articles were quantitative studies: Graham’s [60] cross-sectional survey of mothers who smoked, Michel & Amos’ [53] sociometric analysis of adolescent girls’ peer relationships and smoking, Barraclough’s [61] use of small scale and national survey findings in Indonesia, Morrow et al.’s [62] survey of women factory workers and students in Vietnam, and MacDonald and Wright’s [54] use of cross-sectional survey data from a Canadian high school.

#### The gendered visual culture of women and tobacco

It was notable that over half of the articles on women and smoking – both contemporary and historical – drew from visual analyses and addressed how images of women and smoking have been gendered within media and popular culture, in tobacco advertising, and also in tobacco prevention campaigns. As confirmed by Tinkler [45,46,48,49] and Cook [47], visual analyses are critical for revealing how smoking has been historically linked to gender and class, and how the cultural symbolism of smoking has changed alongside gains in women’s economic, political, and social status. In this visual context, both the tobacco industry’s use of feminine sexuality to sell cigarettes [24,51] and tobacco control’s “deglamourization” approach to denormalizing smoking as ugly and unattractive for women [41,59] were critiqued, as they communicate a one dimensional or stereotypical presentation of beauty as essential to femininity and women’s identities. These authors explore how dominant social norms have contradictorily positioned women’s smoking as both a source of femininity and sexual attractiveness as well as an unfeminine behaviour (i.e., not “ladylike”), depending upon the historical period, social status and class background of the woman who smokes [41,42,49]. Oaks [44] likewise explored how the body of the pregnant woman and fetal imagery have been mobilized in cessation messaging that intends to evoke women’s guilt and shame (i.e., as “bad” mothers) about the health harms that smoking causes.

#### Understandings of gender

In the majority of items, the approach to gender in relation to smoking is implicit, in that it was not explicitly theorized but linked broadly to a feminist or woman-centered stance that prioritizes gender equality. In this context, women’s empowerment was seen as “freedom from smoking.” This runs counter to the gender-based tactics of the tobacco industry, wherein the “freedom to smoke” has been mobilized within cigarette advertising as an act of women’s liberation, intended to symbolize their equality with men [42]. Young women were seen as particularly vulnerable to cultural messages and to media messaging that aligns smoking with a sophisticated and/or fashionable identity, or as an expression of heterosexual femininity [43,53,55].

In several works, feminine gender identities or norms around smoking were seen as complex and socially contingent, suggesting that perceptions of smoking as a feminine identity are changeable, bound up in the broader cultural representations of women’s smoking and to patterns of gendered consumption. These items took a critical view of attempts to exploit gender-based differences and to the objectification of femininity and women’s bodies and sexuality. This type of analysis rests upon an assumption that gender identities are socially constructed and linked to social power differentials, and seeks to delink essentialist notions of femininity and women’s identities. Explicit uses of gender theory/theorists were seen in about one-fifth of the articles in this category and included: Bourdieu’s masculine domination, symbolic violence and feminine appearance imperatives [59,63], Butler’s gender performativity [50,55], and Connell’s emphasized femininity [58].

#### Implications for interventions

In the collection of items in this category, there were no research studies that pilot- or systematically-tested a women-specific or women-sensitive TRC intervention. In one sense, providing a detailed account of women’s smoking might be considered a type of cultural or historical intervention, in that such social science scholarship addresses the invisibility of women from the research literature and critically interrogates sexism and gender stereotyping. It might also be inferred from the feminist or woman-centered approach seen within much of this scholarship, that there is a need to engage women in TRC in ways that are positive or empowering for gender – primarily by focusing on smoking cessation as a women’s health issue in its own right, above and beyond the domains of pregnancy or mothering [41,44]. In addition to the need to challenge portrayals of smoking as glamorous or “sexy” feminine practice by Hollywood films or in tobacco advertising [49,50,55], scholarship addressing the social history of women and smoking also makes it clear that there have been contradictory or “mixed” messages about smoking, gender and femininity within visual and popular culture. As such, there is a need to think critically about how non-smoking has been linked to women’s attractiveness and prioritize beauty over health in anti-tobacco campaigns directed towards women [41,49]. Finally, researchers addressing tobacco interventions at the structural level also made a strong argument for considering the social context of gender-related influences on smoking as they intersect with poverty and social disadvantage, and the broader conditions of women’s lives [60,62]. As argued by feminist researchers such as Oaks [44], even “positive” gender-specific anti-tobacco messages that are geared towards empowering women to make “better” health decisions can have the moralistic effect of “blaming the victim,” in that they emphasize health as personal/individual responsibility and choice. Indeed, as was first argued by Greaves [41] almost two decades ago, “It is too easy to think of women smokers as simply agents of their poor health or instruments of their own addiction”, leading to a “sexist and disrespectful approach” to tobacco control policy and programming (p. 120). As such, “the challenge is to appeal to women and girls who smoke by using methods that do not blame them” [44], in designing messaging and tobacco interventions that are “woman-specific” *and* “woman-positive”, recognizing the complexity of tobacco use in women’s lives beyond a psychosocial or behavioural approach [41].

#### Gaps

Only two of the articles in this sub-category addressed gender-related factors and smoking by women outside of Western contexts, in countries including Vietnam [62] and Indonesia [61]. In their commentary and historical review, Amos and Haglund [42] also made a strong case in support of a 21^st^ century tobacco control approach that is more global in its gender focus, citing the fact that women in the developing world are vulnerable to growing rates of smoking due to shifts in gender roles and targeting from the tobacco industry.

In addition to the absence of accounts of women’s experiences with tobacco and cessation outside of North America and the United Kingdom, the gender and tobacco research literature might benefit from intersectional analyses of gender and smoking as advocated by contemporary feminist theories of health. This would include emphasis on interventions that recognize diversities within gender, and that consider how gender intersects with other identity categories, social factors and/or systems of inequity (e.g., sexuality, race, socioeconomic status). Most notable, however, is that despite decades of descriptive research focussed on the links between women, femininity, and smoking, intervention efforts have not responded adequately by incorporating this knowledge in women-centered cessation programs.

### Men’s smoking and gender identities, roles and norms (n = 21)

#### Description

We identified 21 items focussed on men’s smoking and masculine identities, roles and relations that met the inclusion criteria. All of these articles were published after 2005.

Nine articles explored men’s smoking and fathering in Canada as an aspect of masculine identities. Six articles investigated contemporary or historical constructions of masculinity in American tobacco advertisements and/or lifestyle magazines, such as the Philip Morris Marlboro Man, associated cowboy and Wild West motifs [25,27,64] “new lad” representations of masculinity [65], and an analysis of the cultural and political articulation of cessation by willpower with the male body and masculine ideals of self-control and autonomy [28]. There were four culturally-specific studies of men’s smoking and masculine ideals. For instance, Roberts [66] explored connections between cigarette smoking and masculine role models for Dutch youth in the early 17th century. Korhman [67] questioned the tobacco-related epidemic of men’s deaths in China, invoking a socio-political frame on gender and Asian social pressure to “live the good life” that smoking represents. Focus-group researchers in Java, exploring the values and beliefs that Indonesian teenage boys hold about tobacco, concluded that smoking is a symbol of masculine identity, representing risky behaviour [68]. One group of researchers analyzed how the content in Canadian school textbooks was modified between 1880 and 1960, shifting the representation of masculinity, men and smoking and the associated health risks of tobacco (Cook) [69].

The lone quantitative study in this subsample compared sexual orientation and measures of masculinity and gender conformity among gay men and the general population, concluding that constructs of masculinity predict smoking among both gay and heterosexual men [70]. Finally, Okoli et al.’s [32] systematic review of men-specific tobacco cessation interventions noted how few interventions address the role of masculine ideals and norms in men’s tobacco use.

#### Fathering and tobacco cessation

Six of the nine articles focussed on fathering were empirical findings from qualitative research investigating how ideals of masculinity, such as provider and protector identities [71-73], can conflict with men’s desires to continue an autonomous smoking practice as a “family man.” The longstanding history of interventions that have focussed on mothers and tobacco cessation frames an article addressing the importance of understanding fatherhood as an expression of masculine identity to support men’s cessation efforts [74]. Key program principles to include in interventions for new fathers who smoke are detailed by Oliffe, Bottorff & Sarbit [39]. In an intervention casebook chapter, *Mobilizing Masculinity to Support Fathers to be Smoke-free,* the authors illustrate how positive aspects of men’s masculine identities can be used to assist new dads who are interested in reducing or quitting smoking [38].

#### Understandings of gender

Half of these articles, in particular the articles focussed on fathering, are guided by social constructionist theories of gender and masculinity, as described in the work of Creighton and Oliffe [75]; Kimmel [76]; Courtenay [77,78] and Connell [79,80]. Social constructionist theories of gender view masculinity as social phenomena enacted and maintained by the interplay and performances of individuals and groups within and across social structures. Central to this view of gender and social power, are hegemonic masculinities, the dominant expressions of masculinity which are socially sanctioned at any particular time and locale. Within this framework, cigarette smoking may be theorized as a social reproduction of masculinity or declaration of masculine identity. Because masculinity is socially constructed, it is dynamic and changeable, influenced and taken up in nuanced ways according to various social factors such as culture, race, socio-economic class, age, and sexual identity.

Tobacco use is theorized as a hegemonic masculine activity or response to hegemonic power within this perspective, because smoking fulfills constructed manly ideals of risk-taking, neglect of self-health, and strength and toughness associated with dominant masculinity. Ng et al. [68] and Kohrman [67] make these links between masculine identities and Asian men’s smoking; however, masculinity is ultimately presented as a detrimental practice or pathology that endangers men’s health. Several articles, especially White, Oliffe and Bottorff [27,64,74,81], Cortese and Ling, [65], and to a lesser extent Johnson et al. [82] make theoretical linkages between tobacco as a consumer, cultural commodity, and the use and representation of such commodities in the construction of masculinities. From this perspective, gender becomes co-constructed by individuals’ tobacco consumption and media images in a recurring loop, providing compelling evidence of the need for program interventions to understand the constructed nature and power of gendered imagery.

In contrast, there is evidence for an approach to gender and tobacco cessation among these articles that accesses and amplifies the positive connections between masculine ideals and cessation, rather than vilifying dominant masculinity as a liability. In the context of fatherhood for example, masculine identity then becomes an opportunity to fulfil roles of protector, caregiver and breadwinner, expressing ruggedness and toughness as strong health and well-being, thereby motivating and sustaining smoking cessation [39,64,74].

Pachankis et al. [70] provide the only article under this theme that theorized gender as a psychological construct. The researchers employed multiple measures such as the Boyhood Gender Nonconformity scale, a masculinity Likert scale, and a measure of sexual orientation concealment to compare gay and heterosexual men’s smoking practices, concluding that constructs of masculinity predicted and motivated smoking for both gay and straight men.

#### Implications for interventions

In a comprehensive literature review, Okoli, Torchalla, Oliffe and Bottorff [32] identified men-specific smoking cessation programs, locating 11 studies that delivered interventions to men only. In addition, only 2 of these intervention studies were actually tailored specifically for men [32]. Theoretically, the review positions men, masculinity and tobacco use from the perspective of socially prescribed gender norms, and notes how most tobacco research reports sex differences in interventions, but lacks an understanding of the intersectionality of gender-related factors, and how masculinity is always embedded in a social context, layered and connected to social class, ethnicity, occupation and age factors to influence men’s smoking and cessation.

In regard to creating content for men-specific interventions, the historical analysis of how willpower and the notion of quitting smoking became a presumed masculine virtue is important and practical research [64]. Integrating the philosophical origins of willpower with feminist approaches to the body, and social constructionist perspectives on masculinity, the authors demonstrated how the tobacco industry exploited longstanding gendered assumptions about male power, and men’s presumed ability to easily quit smoking before getting addicted to nicotine. This research is convincing in terms of the need for cessation messaging to skilfully address the deep-seated cultural ideals about men, quitting and willpower.

These articles as a set also underscore the opportunity and need for men-specific tobacco interventions to demonstrate an understanding of gender messaging to the same degree as the tobacco industry. Interventions could incorporate the appeal of masculine ideals into their programming by integrating content about men’s smoke-free identities with references to masculine strength, autonomy, freedom and the ability to take action.

#### Gaps

Most remarkable about this subsample of articles are the limited historical accounts of men’s smoking from the perspective of changing masculine identities over time. This is surprising for two reasons: 1) thorough accounts of women’s smoking and identity exist that have documented the feminization of cigarettes throughout the 19^th^ and 20^th^ centuries and its movement through social classes [41,47,49]; 2) smoking was first taken up in large numbers by men across all cultures; in general, smoking prevalence has always remained higher among men, and the international tobacco epidemic, thus far, has killed far more men than women. That more complete historical accounts documenting men’s smoking culture and masculinity are lacking is perhaps indicative of how well-laminated and entrenched the cultural constructions of men’s smoking with masculinity have remained in our collective understanding. Cook’s [69] analysis supports this view by documenting how regulated and controlled public smoking for men became synonymous with codes of good citizenry and social character, reflecting congruence between school health studies and the messaging in tobacco ads.

### Gender relations and tobacco cessation and reduction (n = 24)

#### Description

Gender relations research refers to the study of the interplay of masculinities and femininities within and between genders [83]. We identified 24 items focussed on gender relations within the context of tobacco cessation or reduction. Over half of these items (n = 14) were studies investigating couple relations and tobacco use in family households. Among these, 8 were also focussed on couple relations and smoking in the context of pregnancy and/or the postpartum. Friendship interactions among young women were the focus of a study that demonstrated how diverse femininities are co-constructed and performed through daily cigarette-related interactions [84]. Two articles compared the gendered ways in which young people perceived smoking [85-87]. Two systematic reviews [88,89], and one book chapter about methods in gender relations research with a case study on couples’ tobacco use [83] focussed on gender relations.

In a NIH Quit Tobacco International initiative article [85], the authors explored men’s smoking and masculinity within the context of Indonesian tobacco advertising, and proposed a cultural intervention based in community gender relations to destabilize tobacco norms. In a critical commentary, Tan [90] denounced the Western emphases on binary categories of feminine and masculine in tobacco research, arguing that such approaches can perpetrate gender ideology and stereotypes in connection with Asia and global smoking trends. He proposed, instead, culture- and location-specific research that examines smoking in the context of polyvalent gender subjectivities along intersecting axes such as class and age.

Two articles were based on the historical research of femininity and masculinity in conjunction with tobacco use patterns. In a longitudinal, quantitative survey of three generations of smokers in Scotland, Hunt, Hannah and West [91] showed how tobacco patterns differed in relation to social class and gender role identity during different periods of the 20^th^ century. In an analysis of cigarette ads within the context of cultural values of the 1880s, Schmitz [92] discussed the shifting gendered meaning of cigarette smoking, arguing that the tobacco industry developed new markets for cigarettes at the end of the 19^th^ century by redressing the long standing associations of cigarettes with women, effeminacy, and urbanism and introducing a more masculinist stance into their advertising content.

#### Gender relations and household smoking

Among the 6 studies investigating barriers to cessation in family households, we found little cohesion in terms of how the researchers approached gender relations [93-98]. The studies explored gender relations in households in Canada, China, Indonesia and Scotland; gender relations were sometimes implicit, and often the analytic category was simply women and men, rather than femininity and masculinity. For instance, Westmaas, Wild and Ferrence [98] in comparing women and men smokers and the role of partner influence on tobacco cessation, found that women were more successful in changing men’s health behaviours than men were in influencing women. The authors proposed that although social network influences may be beneficial for men, they may be perceived as an additional burden by women and as a criticism of how they fulfil their role as a woman and mother. Robinson et al. [97] invoked a gender-based analysis; however, their primary unit was the individual, in-home smoker, and among their sample, the women smokers were compared to the men smokers to derive findings. The authors were careful to distinguish gender from sex factors. They concluded that it was the gendered and class-based role of caring for others, not the sex of the person, which explained women’s indoor smoking, despite bans on home smoking. Although we would argue that Robinson et al. [97] are investigating gender relations within a broad context of social class, their lack of conceptualizing femininity and masculinity also contributed to the strong emphasis on social class factors. Nichter et al. [96] designed their interview-based study with the couple as the unit of analysis (n = 530); however, these researchers also confined their discussion to the gender roles of wives and husbands, or women and men, bypassing gender constructs of femininity and masculinity.

Similarly, disparate approaches to gender relations emerged among the household smoking articles focussed on pregnancy. Male support for pregnant partners and men’s participation in tobacco cessation during pregnancy informed non-theoretical approaches to gender relations in three studies [99-101]. Spousal relations allowed for the investigation of power and control related to tobacco use during pregnancy in three studies [37,102,103]. A grounded theory analysis of interviews with 28 couples yielded distinct typologies that categorized couples’ interaction style related to the women’s tobacco reduction during pregnancy and postpartum [104]. A grounded theory analysis of femininities and masculinities among 27 couples revealed how women may adopt feminine positions as both defenders and regulators of their husband’s smoking [105].

#### Understandings of gender

In many of these studies researchers did not overtly define gender or they communicated ambiguous meanings in regard to gender relations. This collection of items worked with gender from diverse, theoretical perspectives: social constructionism, cultural commodification, feminist and individual health behaviours.

We assessed eight articles as conceptualizing gender from the perspective of individual health behaviours and the interactions of women and men related to tobacco. The authors may have acknowledged the influence of social norms on these health behaviours, but overall gender was not theorized beyond the level of individual behavioural patterns. Six articles employed a feminist lens, emphasising the influence of social class factors in women’s tobacco use, and conducting tobacco research within the context of gender as a factor that was tied to cultural ideologies of power and social power relations. For example, ethnographic research in households in China showed how culturally defined gender roles limited the ability for mothers to create smoke-free homes, and changes related to tobacco use were contained by familial and generational power relations, which could readily become a source of conflict in families with children [93,94].

Two articles [95,105] and a book chapter [83] drew on social constructionist theories of gender [79,106] to explore how femininities and masculinities mapped onto couples’ smoking patterns and reduction efforts. Nichter et al. [85] did not define their theoretical perspective on gender; however, a social constructionist framework was evident in their efforts to affect community-wide tobacco reduction by shifting the smoking identities of young men. Four articles showed how tobacco, as a cultural commodity, was taken up by smokers in the construction of gendered identities, providing a means to socially enact various femininities or masculinities, adhere to gender norms, or reproduce gender ideology [84,86,87,92]. Tan’s [90] commentary called for new tobacco and gender theory, and Nichter et al. [96] did not theorize gender overtly; however, their work suggested a unique approach that we discuss below.

#### Implications for interventions

The integration of knowledge of gender relations and smoking in the design of TRC interventions is at a nascent stage. Efforts to denormalize tobacco use in Indonesia at the household level were described by Nichter et al. [96] in qualitative work with 530 couples. The researchers planned to influence the epidemic of men’s smoking and launch a community-based tobacco cessation movement by encouraging Indonesian women to demand smoke-free homes, promoting awareness and changed tobacco norms at the community level. The underlying theoretical implication here was that gender norms (i.e., men’s reified smoking practices) can be challenged at the local, community level and, if culturally relevant, a gender relations approach to research may be helpful for devising effective interventions for entire communities. Nonetheless, enlisting Indonesian women to promote smoke-free masculine identities, or purposely linking young men’s popularity to the approval of smoke-free women [85] is a controversial strategy from the perspective of feminist politics in the West, as well as potentially loading responsibility for initiating change onto women’s shoulders with unknown risks. This approach is not transformative (i.e., improving gender equity as well as health), as it perpetuates women’s responsibility for health management and does not shift gender norms [107,108]. However, the importance of messaging young men differently than mature men [85] is an important conceptual tool for practitioners creating interventions, and implies a social constructionalist stance that views gender identity as plural and changeable and intersecting with multiple factors such as age and social location.

By employing social constructionist gender theories [79,106], parallel research with couples in Canada identified how women adopted specific femininities in regard to men’s smoking [105] and how parenting styles and femininities and masculinities mapped onto tobacco reduction and cessation efforts [95]. In gender relations research with pregnant women and their partners, the ways couples responded to women’s efforts to reduce or stop smoking depended on the couples’ established interaction patterns with respect to tobacco (i.e., disengaged, conflictual, or accommodating styles) [104]. As a result, the authors recommended de-linked, women-centered, couple-informed TRC interventions for pregnant women [37,104]. These findings were also translated into an intervention booklet for women and distributed online and in community clinics [109].

#### Gaps

We were unable to identify interventions in the literature that have incorporated a gender relations approach and knowledge of femininities and masculinities into tobacco cessation programming for people who are not parenting or living in family households, perhaps reflecting a long held concentration on tobacco use and reproduction, especially for women, to the exclusion of addressing individuals as women (or men). An intergenerational approach to gender relations and tobacco use would also be beneficial to refocus interventions beyond the context of the heterosexual nuclear family.

### Gender issues in tobacco control policy (n = 10)

#### Description

Ten articles identified by our review addressed gender issues at the broader, structural level of tobacco control policy. Seven of these articles focussed on gender issues in women and girls’ tobacco use, and represented relatively newer additions to the field, published in the years 2003–2012.

The gender- and policy-focussed items we retrieved fell into three categories: articles that inferred policy implications from empirical studies of specific or comparative tobacco policy contexts [110,111]; review articles that provided summary and critical analysis of policy-relevant research through a gender lens [112-114]; and manuscripts that provided a more general description of current policy gaps and options [29,115-117]. However, recommendations towards the development of tobacco control policies that addressed gender issues cut across all of these categories.

#### Understandings of gender

The policy-focused literature for the most part contained clear and explicit definitions of gender, clearly distinguished gender from sex, and prioritized the complex interactions between sex and gender as they influence tobacco use. In addition to citing the need for better prevalence and policy data on smoking that is disaggregated by gender and sex, policies were critiqued for adopting a “gender blind” or “one size fits all” approach to tobacco control, when there is a need for gender-specific or gender-sensitive approaches [29,111] that contextualize gender beyond the level of individual identity, role, or relational influences, as a broader “upstream” or macro-level social determinant of health. In this context, gender was placed on a continuum (as opposed to a male/female binary) and was one of several intersecting categories that influenced smoking [29]. Finally, Morrow and Barraclough [117] made a compelling argument for policy informed by a theory of gender as socially constructed, and argued that within the tobacco policy literature “gender” has typically implied women and girls, with the influence of men’s gender and masculinities on smoking conspicuously absent.

#### Implications for interventions

Policy-focussed articles underscore the need to advocate for changes to broader social and economic structures and to redress gender-related inequities through implementing gender-sensitive tobacco control policies [117]. Recommendations towards a gender-sensitive policy framework range from refining measurement tools to ensure the collection of sex and gender data in policy research, to engaging with gendered subpopulations in collaborative, participatory policy development (i.e. beyond community consultation) [118]. There is also consideration of how gender-specific policies might work towards a broader aim of social justice, through transforming gender inequities (empowering women socially and economically) as opposed to accommodating gender (playing to women’s roles as nurturers or mothers) or exploiting gender (paternalistic approaches that “protect” women from smoking) [116].

In this literature, there is also critical consideration of the “unintended” – and often decidedly gendered – consequences of adopting particular policy interventions [113]. For example, Greaves and Hemsing [114] have argued that measures such as price and taxation increases create an undue burden on subpopulations of women with higher smoking prevalence such as low-income and lone-parent mothers, without the corresponding supports and programming for cessation. Likewise, Burgess et al. [112] provide review evidence to support their argument that tobacco control policies designed to protect children from second-hand smoke have the unintended effect of stigmatizing their mothers, blaming an already socially marginalized group of women, and perhaps worsening their health status. As the policy-focused work makes clear, it is vital to consider gender-related factors in relation to socioeconomic status, racialization, mental health, and sexual minority statuses, because they can be associated with an increased vulnerability to tobacco use and with fewer reductions in smoking prevalence from policy measures [113,114,118]. As gender-related factors differ by social and cultural context, policy solutions also need to vary according to localized gender-based norms and cultural variations in social roles for women and men [110,111,117]. The analyses of women and tobacco policy advocate for what they term “gender and diversity” analysis that considers gender as it intersects with other social categories [116,118].

#### Gaps

To date, comprehensive or systematic analyses of tobacco policies and their potentially differential effects by sex and gender have been few. Clearly, the policy-focussed literature on men, masculinities and tobacco would benefit from greater attention and development. Additionally, as the current policy literature is situated within the Western or post-industrial context, greater attention needs to be afforded to gender and tobacco policy in developing countries where smoking rates are rising and the “first and second waves” of the tobacco epidemic are in progress [115-117].

### Principles and recommendations

In summary, after incorporating these analyses of various groups of literature related to gender and tobacco, we developed three key principles and recommendations to guide the inclusion of gender-related factors when developing tobacco reduction and cessation interventions.

### Principle 1: Tobacco interventions need to be built upon solid conceptualizations of gender

Recommendation: All too often, gender has been absent from the research literature on smoking cessation, and when it is present gender remains poorly defined. To advance TRC interventions, we recommend that tobacco researchers adopt a theoretical framework that accounts for gender in the following ways: 1) gender is relational and dynamic; 2) gender identities are diverse and fluid; and 3) gender is shaped by social context and its interaction with other social determinants of health (e.g. age, rurality, socioeconomic status, race and ethnicity). For example, interventions that consider gender and its intersection with social and/or economic disadvantage may have increased effectiveness and relevance to specific populations of women and men, rather than assuming relevance based on gender alone. Theorizing gender in these ways adds definitional clarity, assists in distilling gender from sex, acknowledges the cultural and temporal nature of gender, and promotes a view of gender in relation to other social influences on tobacco use and cessation.

### Principle 2: Tobacco interventions must include components that comprehensively address gender-related influences

Recommendation: Tobacco researchers in both women’s and men’s health have called for gender-specific and gender-sensitive interventions to better accommodate the needs of subgroups of individuals. Gender-sensitive interventions are those that prioritize gender-related influences or needs within the context of an intervention delivered to both men and women. Gender-specific interventions are designed specifically, or only for, men or women. In the past, sex and gender-specific interventions have been designed primarily for pregnant women; however, when we conceptualize gender as it intersects with other social factors such as age, ethnicity, sexual identity, disadvantage and social class, or relationship and marital influences, we find the opportunity to improve tobacco interventions by tailoring them for more specific and diverse audiences of smokers.

An example of a gender-specific intervention that does not stigmatize pregnant women who smoke can be found in the booklet, *Couples and smoking: What you need to know when you are pregnant* [109]. This woman-centred approach to reduction and cessation is based in gender-sensitive research that has identified couple interactions and couple typologies that emerge when women who smoke become pregnant, removing the focus from initiating change in the woman’s behaviour to the social context of tobacco in pregnancy and couple relations.

### Principle 3: Tobacco interventions should promote gender equity and healthy gender norms, roles and relations

Recommendation: In order to counter the exploitative approach to gender in tobacco industry marketing and promotion campaigns, we recommend an approach to gender identities and gender relations that is based on principles of equity and empowerment. For example, interventions seeking to address gender should consider how alignment with particular gender identities and constructs of femininity and masculinity influence cessation and/or continued smoking. As examples, this could include notions of masculinity and “cold turkey” quitting methods, and the perceived links between femininity and sexual attractiveness, rebelliousness or independence. A gender-sensitive approach to interventions would generate or acknowledge positive representations of masculinity and femininity in countering the stereotypes the tobacco industry continuously perpetrates, even if they are not the most popular representations.

An example of a gender-specific intervention that does not promote negative representations of femininity or masculinity is available in the booklet *The Right Time, The Right Reasons: Dads talk about reducing and quitting smoking* [119]*.* This material is based on gender research with fathers who smoke and assists their desire to quit by appealing to masculine ideals such as strength, decisiveness, and being healthy for one’s family.

Gender role research can be challenging because of its tendency to pre-determine norms and therefore reinforce static, conventional notions of gender [11]. Contemporary constructivist gender theories grounded in performativity and plural masculinities and femininities provide an inductive means for building targeted TRC interventions [80,120]. Related to this, when designing interventions there is a need to be cognizant of the local values of sub-groups and their influence on gendered practices in the context of tobacco use. Current approaches to gender and health theory acknowledge that gender “depends” on other social categories and practices for its meaning and through these intersections create inequalities and power relations that affect both women and men [121,122]. These considerations need to be taken into account in understanding smoking trends and the way they intersect with other social factors including regional and global gendered ideals and structural controls as a means to promoting gender equity among and across men and women.

### Strength and limitations of method

Conducting this scoping review enabled us to provide a synthesis of the emergent field of gender-related influences in TRC and propose how these influences might be integrated in smoking interventions. Highlighted are rich gendered contexts to extend sex differences research and afford nuanced understandings about the need for targeted TRC interventions. The strength of scoping review methods resides in its capacity to capture the essence of an emergent body of knowledge amid making recommendations for how best to build upon and apply those understandings. In addition, the scoping review neatly fits with the ontological and epistemological frames of social constructionist theories wherein the interpretive and iterative nature of the findings drawn from the current scoping review relay and reflect gender as diverse but with prevailing patterns embedded in social structures and power dynamics. In terms of limitations, by scoping gender-related influences only sex influences on tobacco use are less visible. To address this future research might integrate sex and gender to further develop targeted TRC interventions.

## Conclusion

This scoping review demonstrates the wide range of approaches to researching gender and smoking cessation. By considering the gender literature on women and smoking and men and smoking side-by-side, we have identified important gender gaps in knowledge as well as some strategies toward improving future TRC interventions.

This knowledge and evidence for how masculinities, femininities and the interplay of those conventions shape and are shaped by smoking practices are key to the effectiveness of TRC interventions. Integrating sex and gender, and advancing gender to explicitly include social factors including class, race and culture will go some distance in achieving health equity and empowerment along with tobacco reduction, cessation and prevention. While improving the way in which gender is *integrated* into TRC interventions and tobacco control literature more widely is necessary, it is not sufficient for ensuring or improving gender equity and reducing health inequities. Indeed, contemporary thinking in gender and tobacco control articulates the goal of doing gender transformative work [107,108]: that is improving health and gender equity *at the same time* in intervention or policy design, thereby taking responsibility for not just acknowledging gender, but rather, shifting gender and its enactment, effects, performance and meaning, in the context of tobacco reduction or cessation.

## Endnotes

^a^While this scoping review is focused on gender, we pay close attention to the interplay between gender *and* sex, in regards to how the biological sciences distinguish sex by various anatomies, physiologies, genes and hormones (Johnson & Repta) [23]. We employ the distinctions of gender and sex with the awareness that such categories are intimately connected to social and cultural ideologies of power, in constant negotiation, often artificially dichotomous, and often conceptually elusive. Therefore, key to our work is the knowledge that sex and gender are continua and change across time and history and vary within and between individuals and groups. Acknowledging and anticipating both sex and gender as operating outside of female–male binaries in dynamic ways reminds us that smoking patterns and cessation efforts vary across time and within subgroups of women and men.

^b^Gender specific interventions are those designed exclusively for women or men. Gender-sensitive interventions can be designed for women or men, but are sensitive to how the approach and outcomes may be influenced by gender-related factors.

^c^It is notable that close to half (45% or 36/80) of the total items were published by an author of this article and/or a member of the Investigating Tobacco and Gender (iTAG) research team.
